# Impact of sex differences on cardiac injury in critically ill patients with COVID-19

**DOI:** 10.1186/s12931-023-02581-5

**Published:** 2023-11-20

**Authors:** Mathieu Jozwiak, Denis Doyen, Pierre Denormandie, Antoine Goury, Jonathan Marey, Frédéric Pène, Alain Cariou, Jean-Paul Mira, Jean Dellamonica, Lee S. Nguyen

**Affiliations:** 1 Service de Médecine Intensive Réanimation, Hôpitaux Universitaires Paris Centre, Hôpital Cochin, Assistance Publique, Hôpitaux de Paris, 27 Rue du Faubourg Saint Jacques, 75014 Paris, France; 2 Université Paris Cité, Paris, France; 3 UR2CA, Unité de Recherche Clinique Côte d’Azur, Université Côte d’Azur, Nice, France; 4 Service de Médecine Intensive Réanimation, Centre Hospitalier Universitaire de Nice, Hôpital L’Archet 1, 151 Rue Saint Antoine de Ginestière, 06200 Nice, France; 5 Service de Médecine Intensive Réanimation, Centre Hospitalier Universitaire de Reims, Rue du Général Koenig, 51092 Reims, France; 6 Unité de Soins Intensifs Pneumologiques, Hôpitaux Universitaires Paris Centre, Hôpital Cochin, Assistance Publique, Hôpitaux de Paris, 27 Rue du Faubourg Saint Jacques, 75014 Paris, France; 7 Recherche et Innovation, Groupe hospitalier privé Ambroise Paré, Hartmann, 48Ter Bd Victor Hugo, 92200 Neuilly-Sur-Seine, France

**Keywords:** Cardiac injury, COVID-19, Echocardiography, Electrocardiogram, Outcomes

## Abstract

**Background:**

COVID-19 infections are associated with accrued inflammatory responses which may result in cardiac injury. Immune response to infection appears different between men and women, suggesting that COVID-19 patients’ outcomes may differ according to biological sex. However, the impact of biological sex on the occurrence of cardiac injury during intensive care unit (ICU) stay in COVID-19 patients remain unclear.

**Methods:**

In this multicenter and prospective study, we included consecutive patients admitted to ICU for severe COVID-19 pneumonia, during the first two pandemic waves. Biological, electrocardiogram (ECG) and echocardiographic variables were collected on ICU admission. Cardiac injury was defined by increased troponin above 99th percentile of upper norm value and newly diagnosed ECG and/or echocardiographic abnormalities. The primary endpoint was the proportion of patients with cardiac injury during ICU stay according to biological sex. The impact of biological sex on other subsequent clinical outcomes was also evaluated.

**Results:**

We included 198 patients with a median age of 66 (56–73) years, 147 (74%) patients were men and 51 (26%) were women. Overall, 119 (60%) patients had cardiac injury during ICU stay and the proportion of patients with cardiac injury during ICU stay was not different between men and women (60% vs. 61%, p = 1.00). Patients with cardiac injury during ICU stay showed more cardiovascular risk factors and chronic cardiac disease and had a higher ICU mortality rate. On ICU admission, they had a more marked lymphopenia (0.70 (0.40–0.80) *vs.* 0.80 (0.50–1.10) × 10^9^/L, p < 0.01) and inflammation (C-Reactive Protein (155 (88–246) *vs.* 111 (62–192) mg/L, p = 0.03); D-Dimers (1293 (709–2523) *vs.* 900 (560–1813) µg/L, p = 0.03)). Plasmatic levels of inflammatory biomarkers on ICU admission correlated with SAPS-2 and SOFA scores but not with the different echocardiographic variables. Multivariate analysis confirmed cardiovascular risk factors (OR = 2.31; 95%CI (1.06–5.02), p = 0.03) and chronic cardiac disease (OR = 8.58; 95%CI (1.01–73.17), p = 0.04) were independently associated with the occurrence of cardiac injury during ICU stay, whereas biological sex (OR = 0.88; 95%CI (0.42–1.84), p = 0.73) was not. Biological sex had no impact on the occurrence during ICU stay of other clinical outcomes.

**Conclusions:**

Most critically ill patients with COVID-19 were men and experienced cardiac injury during ICU stay. Nevertheless, biological sex had no impact on the occurrence of cardiac injury during ICU stay or on other clinical outcomes.

*Clinical trial registration* NCT04335162

**Supplementary Information:**

The online version contains supplementary material available at 10.1186/s12931-023-02581-5.

## Background

Since December 2019, a worldwide pandemic of Coronavirus disease (COVID-19) due to the emerging coronavirus SARS-CoV-2 has been observed from China [1]. Although most patients are asymptomatic or developed a non-severe form of COVID-19, most severe patients require to be admitted to intensive care unit (ICU) because of hypoxemic acute respiratory failure, [2–4]. Besides acute respiratory distress syndrome (ARDS), critically-ill patients with COVID-19 may develop cardiac injury [5–12], kidney injury [13–16], thrombotic complications [17, 18] or disseminated intravascular coagulation [19, 20]. Nevertheless, cardiac injury was defined in most studies only by an increase in troponin, which does not necessarily reflect cardiac injury and is likely to be multifactorial [21], regardless of new abnormalities in electrocardiogram (ECG) and/or echocardiography [22] and studies including ECG and/or echocardiographic abnormalities to define cardiac injury did not include critically ill patients [23] or did not describe ECG and echocardiographic abnormalities at all or in a very basic way [2, 3, 12].

Previous studies have reported that biological sex may influence the severity of COVID-19 and patients’ prognosis and clinical outcomes [19, 20, 24–26]. Higher rates of acute kidney injury, cardiac injury and disseminated intravascular coagulation were retrospectively found in men [19, 20], men had a higher risk of developing a severe form of COVID-19 [26] and men had a higher risk for mortality [20, 24–26] and infections [25] than women. However, these previous studies were retrospective [19, 20] and/or did not specifically focus on critically ill patients [19, 20, 24–26]. We conducted this multicenter prospective and observational study to assess the impact of biological sex on the occurrence of cardiac injury and other clinical outcomes during ICU stay in critically ill patients with COVID-19.

## Methods and patients

This multicenter, prospective and observational study was conducted in three intensive care units (ICUs) and one respiratory medicine department of French University hospitals and was approved by the Ethics committee of Nice hospital (number R04-022 3313140420). Informed consent was waived but all patients or next of kin were informed about the study. The study complied with the PRICES statement [27] and the Strengthening the Reporting of Observational Studies in Epidemiology (STROBE) statement guidelines [28].

### Patients

We included all consecutive patients with COVID-19 aged 18 years or older admitted to participating units during the first two pandemic waves. Due to the local organization of care during the pandemic, the respiratory medicine department was transformed into ICU to admit critically ill patients and manage ventilated patients. All patients had a positive real-time reverse transcriptase-polymerase chain reaction assay for SARS-CoV-2 in nasal swabs. Exclusion criteria were patients with poor echogenicity, defined as the inability to correctly align the Doppler beam to obtain reliable Doppler measurements and/or to correctly delineate the endocardium for measuring the left and right ventricular end-diastolic area (LVEDA and RVEDA), and patients with a decision to withdraw life-sustaining therapy.

### ECG analyses and echocardiographic measurements

A transthoracic echocardiography (TTE) examination with concomitant 12- or 18-lead ECGs was performed in all patients within the first 24 h of ICU admission. ECGs were interpreted by the same experienced cardiologist and TTE measurements were performed by experienced board-certified operators. All echocardiographic measurements were performed using a Philips CX 50 (Philips Healthcare, DA Best, The Netherlands) or a Vivid E9 (GE Healthcare, Horten, Norway) at end-expiration following the current recommendations [29]. TTE measurements were averaged on three consecutive end-expiratory measurements in patients with sinus rhythm and five consecutive end-expiratory measurements in patients with atrial fibrillation [30]. All contours were hand-drawn. All ECGs analyses and TTE measurements were performed offline blinded to patients’ identities.

ECG signs of left ventricular (LV) hypertrophy were defined as follows: a Sokolow–Lyon index > 35 mm, and/or a R in aVL > 11 mm, and/or a Cornell voltage duration product > 2440 mm.ms and/or a Cornell voltage > 28 mm in men or > 20 mm in women [31]. ECG signs of right ventricular (RV) strain were defined as follows: T waves inversion in leads V1-V4, and/or a QR pattern in V1, and/or a S1Q3T3 pattern, and/or an incomplete or complete right bundle branch [32].

Long-axis and short-axis parasternal views as well as apical five, four, three and two-chamber views were recorded. From the apical five- and four-chamber views, we measured: the left atrial area, the early (E) and atrial (A) peak velocities of the mitral flow with pulsed Doppler, the early diastolic (e’) peak velocity of the lateral and septal mitral annulus with Tissue Doppler Imaging, the tricuspid annular plane systolic excursion, the systolic tricuspid annular velocity with Tissue Doppler Imaging, the tricuspid regurgitant jet velocity and the LV and RV end-diastolic areas (LVEDA and RVEDA). From these variables, we calculated e’ _averaged_, the E/A, E/e’_averaged_ and RVEDA/LVEDA ratios. The LV ejection fraction and left atrial volume were calculated by the modified Simpson’s rule. The systolic pulmonary artery pressure was estimated from the tricuspid regurgitant jet velocity. The LV mass was estimated from LV linear dimensions acquired from 2D long-axis parasternal view as recommended. LV hypertrophy was defined by a LV mass > 95 g/m^2^ in women and > 115 g/m^2^ in men [29].

LV systolic dysfunction was defined by a LV ejection fraction < 50% [29]. LV diastolic dysfunction was defined as the presence of at least two of the following abnormalities: abnormal e’-wave velocity (e’_septal_ < 7 cm/s or e’_lateral_ < 10 cm/s), increased E/e’ ratio (E/e’_averaged_ ratio > 14 in patients with sinus rhythm or E/e’_septal_ ratio > 11 in patients with atrial arrhythmia) or left atrial dilation (left atrial volume > 34 mL/m^2^) [33]. RV systolic dysfunction was defined by at least one of the following criteria: tricuspid annular plane systolic excursion < 17 mm and/or systolic tricuspid annular velocity < 9.5 cm/s and/or RV fractional area change < 35% [29]. Cor pulmonale was defined by a RVEDA/LVEDA ratio > 0.6 with a paradoxical septal motion [34].

### Patient management

Ventilatory support in the different ICUs included high-flow nasal oxygen therapy, non-invasive ventilation or invasive mechanical ventilation, based on the severity of respiratory failure and local protocols. Mechanically ventilated patients were placed in 45-degree semi-recumbent position and ventilated using volume assist-controlled mode. Neuromuscular blocker agents and prone positioning were used based on current recommendations in non-COVID-19 patients with ARDS [35]. The compliance of the respiratory system was calculated as tidal volume/(plateau pressure − total positive end-expiratory pressure). The driving pressure was calculated as plateau pressure − total positive end-expiratory pressure. Administration of corticosteroids and tocilizumab followed the national guidelines during the study period [36, 37].

### Data collection and endpoints

Patient characteristics, biological variables as well as ECG and echocardiographic variables were collected on ICU admission. Clinical outcomes were collected during ICU stay with a maximum follow up at ICU discharge or death, whichever occurred earlier.

The primary endpoint was the proportion of patients with cardiac injury during ICU stay according to biological sex. Secondary endpoints were the proportion of patients with ARDS, acute kidney injury or disseminated intravascular coagulation during ICU stay according to biological sex and the ICU mortality rate according to biological sex.

Cardiac injury was defined by an increase in high-sensitivity troponin T or troponin I levels above its 99th percentile (14 ng/L for troponin T and 40 ng/L for troponin I) and newly diagnosed ECG and/or TTE abnormalities, with ECG and TTE abnormalities considered as newly diagnosed if unknown before ICU admission [5]. Newly ECG abnormalities considered for the definition of cardiac injury were the following: (i) ECG signs of LV abnormalities including repolarization abnormalities involving at least two contiguous leads in the same territory, pathological Q waves in at least two contiguous leads, newly diagnosed left bundle branch block, life-threatening ventricular arrhythmia, or severe brady-arrhythmia, (ii) new-onset atrial arrhythmias and (iii), signs of RV strain. Newly TTE abnormalities considered for the definition of cardiac injury were the following: (i) echocardiographic signs of LV abnormalities including LV systolic dysfunction or wall motion abnormalities, (ii) LV diastolic dysfunction, (iii) cor pulmonale, (iv) RV systolic dysfunction, (v) any significant valvulopathy or (vi) pericardial effusion.

ARDS was defined according to the Berlin definition [38], acute kidney injury according to the Kidney Disease Improving Global Outcomes classification [39] and disseminated intravascular coagulation according to current guidelines [40–42].

### Statistical analyses

Continuous variables were expressed as median (interquartile range), due to non-normal distribution of most variables, as assessed by a Shapiro–Wilk test and categorical variables were expressed as numbers (percentages). Between groups comparisons were performed using Mann–Whitney tests for continuous variables and Pearson’s Chi-square or Fisher exact tests for categorical variables. Correlations between continuous variables were tested using non-parametric Spearman correlations, due to non-normal distribution. Risk factors on ICU admission for the occurrence of cardiac injury during ICU stay were identified by a logistic regression model and results were given as odds ratio (OR) with their 95% confidence interval (CI). The variable biological sex was forced into the model as the main goal of the study was to assess the impact of biological sex on the occurrence of cardiac injury during ICU stay. All the other variables included in the model were variables with clinical relevance found to be significantly associated with cardiac injury with a p-value < 0.20 at univariate analysis. All significant variables with collinearity were excluded from the regression model. The percentage of missing data for each variable is detailed in Additional file 1: Table S1. Descriptive statistics were only carried out on the available data. Missing values for covariates included in the multivariable model were handled by multiple imputations with chained equation [43]. Statistical analysis was performed with SPSS 23.0 software (IBM, Armonk, USA). All tests were two-sided and a p-value < 0.05 was considered statistically.

## Results

### Study population

Among the 232 patients admitted to the different participating centers during the study period (March 2020 to March 2021), 26 (11%) were excluded due to poor echogenicity and 8 (3%) due to decision to withdraw life-sustaining therapy. Overall, 198 patients were included: the median age was 66 (56–73) years, 157 (79%) had cardiovascular risk factors, 22 (11%) had immunosuppression and no patients were vaccinated against SARS-CoV-2. The median delay from onset of symptoms to ICU admission was 9 (6–11) days, corticosteroids and tocilizumab were respectively administered in 171 (86%) and 46 (23%) patients and average ICU mortality rate was 22% (Table 1).

**Table 1:** Characteristics, management and outcomes of patients with and without cardiac injury during ICU stay

Variables	No cardiac injury (n = 79)	Cardiac injury (n = 119)	p value
Clinical characteristics			
Age (years)	63 (52–69)	70 (61–75)	< 0.001
Male gender, n (%)	59 (75)	88 (74)	1.00
SAPS-2	33 (24–43)	45 (37–65)	< 0.001
SOFA score on ICU admission	5 (3–9)	7 (3–11)	0.04
Body mass index (kg/m^2^)	28 (26–32)	28 (26–31)	0.48
Obesity, n (%)	35 (44)	43 (36)	0.30
Arterial hypertension, n (%)	27 (34)	76 (64)	< 0.001
Diabetes mellitus, n (%)	20 (25)	41 (35)	0.21
Dyslipidemia, n (%)	15 (19)	37 (31)	0.07
Coronary artery disease, n (%)	4 (5)	20 (17)	0.01
Stroke, n (%)	0 (0)	9 (8)	0.01
Smokers, n (%)	10 (13)	23 (19)	0.25
Cardiovascular risk factors, n (%)	56 (71)	101 (85)	0.01
Chronic cardiac disease, n (%)	1 (1)	13 (11)	< 0.01
Chronic respiratory disease, n (%)	5 (6)	6 (5)	0.76
Chronic kidney disease, n (%)	3 (4)	23 (19)	< 0.01
Neoplasia, n (%)	9 (11)	20 (17)	0.31
Immunosuppression, n (%)	3 (4)	19 (16)	0.01
Renin-Angiotensin System Blockers, n (%)	20 (25)	55 (46)	< 0.01
Treatments on ICU admission			
Corticosteroids, n (%)	71 (90)	100 (84)	0.29
Tocilizumab, n (%)	20 (25)	26 (22)	0.61
Antiviral drugs, n (%)	2 (2)	9 (8)	0.21
Low-dose thrombophylaxis, n (%)	7 (9)	10 (8)	1.00
Enhanced intermediate-dose thrombophylaxis, n (%)	64 (81)	74 (62)	< 0.01
Curative anticoagulation, n (%)	8 (10)	44 (37)	< 0.001
Management during ICU stay			
Neuromuscular blocker agents, n (%)	42 (53)	83 (70)	0.02
Prone positioning, n (%)	40 (51)	74 (62)	0.14
Venovenous ECMO, n (%)	3 (4)	5 (4)	1.00
Renal replacement therapy, n (%)	4 (5) (55)	32 (27)	< 0.001
Delays and outcomes			
From onset of symptoms to ICU admission (days)	9 (7–11)	8 (6–11)	0.24
Acute respiratory distress syndrome, n (%)	46 (58)	86 (72)	0.05
Pulmonary embolism, n (%)	8 (10)	21 (18)	0.16
Acute kidney injury, n (%)	19 (24)	72 (60)	< 0.001
Disseminated intravascular coagulation, n (%)	0 (0)	1 (1)	1.00
Duration of invasive mechanical ventilation (days)	11 (4–26)	17 (7–36)	0.09
ICU length of stay (days)	9 (5–15)	14 (7–31)	< 0.01
ICU mortality rate (n, %)	7 (9)	36 (30)	< 0.001

n = 198. Data are expressed as median (interquartile range) or numbers (percentages)

ECMO: extracorporeal membrane oxygenation; ICU: intensive care unit; SAPS: simplified acute physiology score; SOFA: sepsis-related organ failure assessment

### Cardiac injury during ICU stay

Overall, 119 (60%) patients had cardiac injury during ICU stay (Additional file 1: Figure S1). Patients with cardiac injury during ICU stay were older (70 (61–75) *vs.* 63 (52–69), p < 0.001), had a higher SAPS-2 score (45 (37–65) *vs.* 33 (24–43), p < 0.001) and had more frequently cardiovascular risk factors, coronary artery disease, chronic cardiac disease, chronic kidney disease and chronic medication by Renin-Angiotensin System Blockers than patients without cardiac injury (Table 1). The delay between onset of symptoms to ICU admission did not differ between patients with and without cardiac injury (8 (6–11) *vs.* 9 (7–11) days, p = 0.35) (Table 1). Other clinical characteristics, as well as management and outcomes of patients according to the occurrence of cardiac injury during ICU stay are summarized in Table 1.

**Fig. 1:**
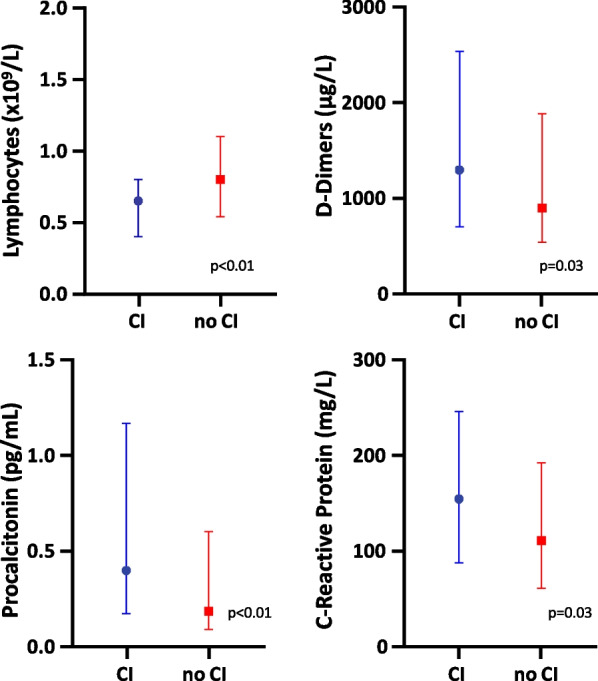
Plasmatic levels of inflammatory biomarkers on intensive care unit admission in patient with (n = 119, blue line) and without (n = 79, red line) cardiac injury (CI) during intensive care unit stay. Variables are expressed as median and interquartile range

**Fig. 2:**
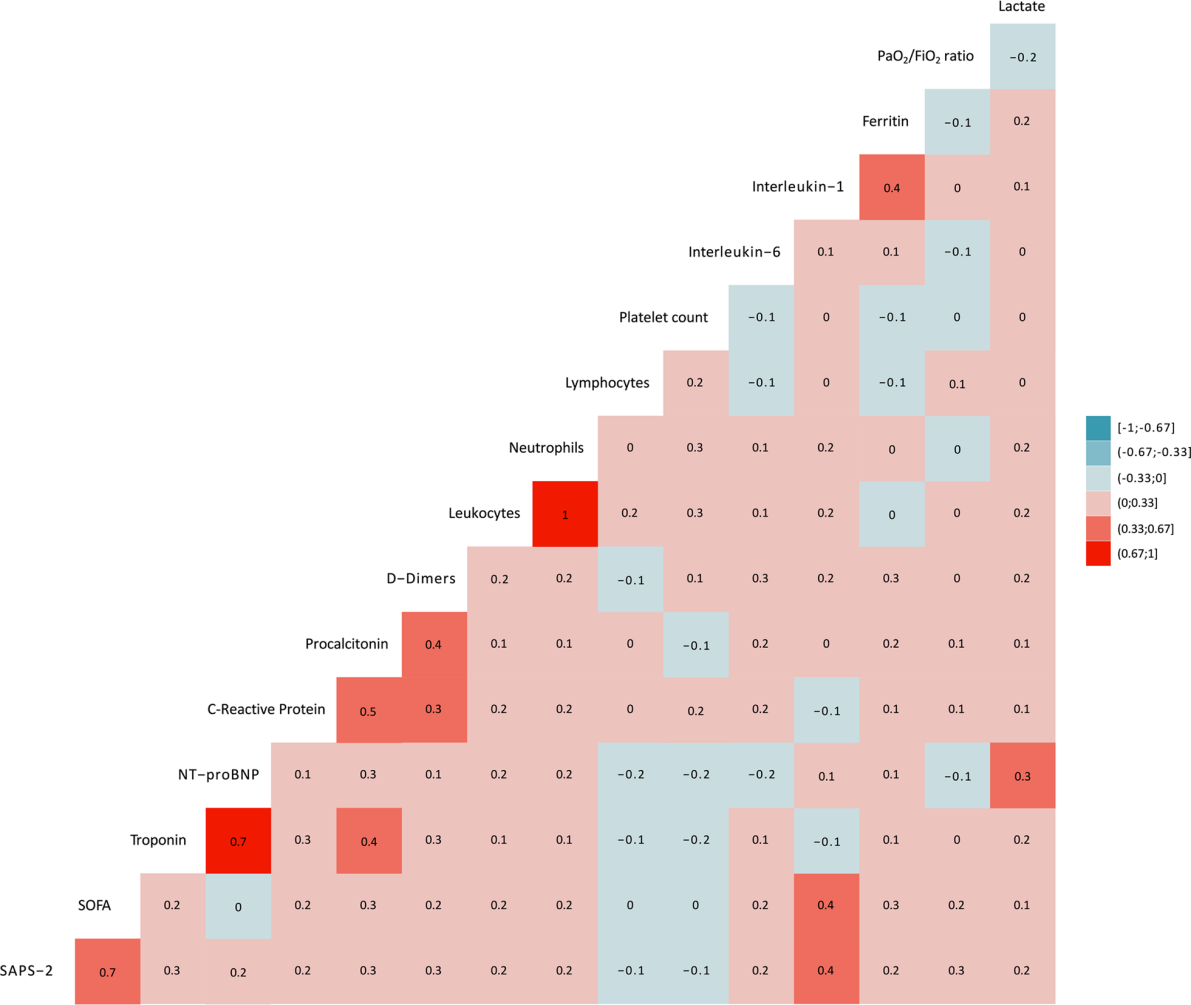
Correlation matrix of clinical severity and plasmatic levels of inflammatory biomarkers on intensive care unit admission. Spearman’s correlations are computed amongst all variables. Positive correlations are represented by red squares and negative correlations by blue squares. Darker colors represent higher correlation coefficient. FiO_2_: inspired oxygen fraction; PaO_2_: partial arterial pressure of oxygen NT-proBNP: N-terminal pro B-type natriuretic peptide; SOFA: sepsis-related organ failure assessment; SAPS: simplified acute physiology score

On ICU admission, patients with cardiac injury had a more marked lymphopenia (0.70 (0.40–0.80) *vs.* 0.80 (0.50–1.10) × 10^9^/L, p < 0.01) than patients without cardiac injury. Plasmatic levels of inflammatory biomarkers were higher in patients with cardiac injury than in those without for D-Dimers (1293 (709–2523) *vs.* 900 (560–1813) µg/L, p = 0.03), C-Reactive Protein (155 (88–246) *vs.* 111 (62–192) mg/L, p = 0.03), procalcitonin (0.40 (0.20–1.20) *vs.* 0.20 (0.10–0.60) ng/L) and platelet, while fibrinogen, ferritin, interleukin-6 and interleukin-1 did not differ between both groups (Table 2, Fig. 1). Both SAPS-2 and SOFA score positively correlated with plasmatic levels of inflammatory biomarkers on ICU admission (Fig. 2).

**Table 2:** Biological variables on ICU admission in patients with and without cardiac injury during ICU stay

Variables	No cardiac injury (n = 79)	Cardiac injury (n = 119)	p value
Leukocytes (× 10^9^/L)	8.5 (6.7–11.2)	9.1 (7.4–12.9)	0.14
Neutrophils (× 10^9^/L)	7.4 (5.3–9.4)	7.6 (5.7–11.7)	0.04
Platelet count (× 10^9^/L)	238 (203–326)	239 (159–291)	0.02
Lymphocytes (× 10^9^/L)	0.80 (0.50–1.10)	0.70 (0.40–0.80)	< 0.01
Fibrinogen (g/L)	6.4 (5.7–7.7)	6.0 (5.1–7.6)	0.32
D-Dimers (µg/L)	900 (560–1813)	1293 (709–2523)	0.03
C-Reactive Protein (mg/L)	111 (62–192)	155 (88–246)	0.03
Procalcitonin (ng/L)	0.20 (0.10–0.60)	0.40 (0.20–1.20)	< 0.01
Ferritin (ng/mL)	1191 (661–1980)	1114 (736–2940)	0.53
Interleukin-6 (pg/mL)	62 (21–126)	66 (22–251)	0.21
Interleukin-1 (pg/mL)	1.70 (0.30–5.90)	1.40 (0.30–9.70)	0.66
Troponin T (ng/L)	10 (12–14)	36 (21–88)	< 0.001
Troponin I (ng/L)	17 (17–23)	27 (17–59)	< 0.01
B-type natriuretic peptide (pg/mL)	36 (20–86)	82 (55–136)	0.001
N-terminal pro B-type natriuretic peptide (pg/mL)	265 (107–622)	1894 (358–5267)	< 0.001
Potassium (mmol/L)	4.0 (3.8–4.3)	4.1 (3.7–4.5)	0.61
Magnesium (mmol/L)	0.90 (0.80–1.00)	0.92 (0.81–1.04)	0.61
Renal clearance (mL/mn)	99 (90–119)	71 (39–93)	< 0.001
Arterial blood lactate level (mmol/L)	1.1 (0.9–1.3)	1.2 (1.0–1.7)	< 0.01

n = 198. Data are expressed as median (interquartile range)

ICU: intensive care unit

On ICU admission, the proportion of patients with LV systolic dysfunction, LV diastolic dysfunction, RV systolic dysfunction, cor pulmonale, significant valvulopathy or pericardial effusion was 14%, 27%, 21%, 3%, 2% and 4% respectively, and the proportion of patients with ECG signs of LV abnormalities, new-onset atrial arrhythmias and signs of RV strain was 38%, 6% and 18% respectively (Table 3). ECG signs of LV abnormalities, new-onset atrial arrhythmias, LV systolic and diastolic dysfunction, RV systolic dysfunction and cor pulmonale were more frequent in patients with than without cardiac injury during ICU stay, while ECG signs of RV strain, significant valvulopathy and pericardial effusion did not differ between the two groups of patients (Table 3). There was no significant correlation between plasmatic levels of inflammatory biomarkers and the different echocardiographic variables on ICU admission (Additional file 1: Figure S2).

**Table 3:** Echocardiographic and electrocardiogram findings on ICU admission in patients with and without cardiac injury during ICU stay

Variables	No cardiac injury (n = 79)	Cardiac injury (n = 119)	p value
Ventilatory management at TTE examination			
High-flow nasal canula oxygen therapy, n (%)	47 (60)	41 (34)	< 0.01
Non-invasive ventilation, n (%)	4 (5)	8 (7)	0.77
Intubation, n (%)	28 (35)	70 (59)	< 0.01
Tidal volume (mL/kg of PBW)	5.9 (5.5–6.1)	6.0 (5.8–6.3)	0.07
Positive end-expiratory pressure (mmHg)	12 (12–15)	12 10–13)	0.02
Driving pressure (mmHg)	13 (10–15)	13 (11–14)	0.58
Respiratory system compliance (mL/cmH2O)	32 (28–40)	30 (25–37)	0.37
Oxygenation variables at TTE examination			
PaO_2_/FiO_2_ ratio	127 (83–167)	121 (83–180)	0.87
PaCO_2_ (mmHg)	37 (33–40)	37 (32–43)	0.51
Hemodynamic variables at TTE examination			
Heart rate (beats per minute)	77 (68–90)	78 (66–90)	0.80
Systolic arterial pressure (mmHg)	122 (114–135)	122 (106–132)	0.38
Diastolic arterial pressure (mmHg)	63 (59–72)	61 (55–72)	0.13
Mean arterial pressure (mmHg)	85 (78–95)	81 (74–93)	0.12
Norepinephrine, n (%)	15 (19)	46 (39)	< 0.01
Norepinephrine dosage (µg/kg/min)	0.10 (0.04–0.25)	0.28 (0.10–0.31)	0.10
Dobutamine, n (%)	0 (0)	1 (0.5)	NA
Dobutamine dosage (µg/kg/min)	0 (0–0)	5 (5–5)	NA
TTE variables			
* LV systolic dysfunction, n (%)*	2 (3)	25 (21)	< 0.001
LV ejection fraction (%)	63 (58–69)	60 (50–66)	< 0.01
VTI (cm)	22 (19–26)	21 (17–25)	0.08
Segmental wall motion abnormality, n (%)	2 (3)	22 (19)	< 0.01
* LV diastolic dysfunction, n (%)*	6 (8)	48 (40)	< 0.001
E/A ratio	0.99 (0.83–1.26)	0.94 (0.76–1.12)	0.09
e’_lateral_ (cm/s)	11.0 (9.1–13.6)	9.0 (7.0–11.0)	< 0.001
e’_septal_ (cm/s)	9.0 (7.6–10.0)	7.1 (6.0–9.0)	< 0.001
E/e’_averaged_	7.3 (6.0–8.6)	8.6 (7.1–10.5)	< 0.001
Indexed left atrial volume (mL/m^2^)	21 (17–29)	23 (16–31)	0.24
LV hypertrophy, n (%)	15 (19)	42 (35)	0.02
Indexed end-diastolic LV volume (mL/m^2^)	45 (35–52)	44 (34–58)	0.59
* RV systolic dysfunction, n (%)*	6 (8)	36 (30)	< 0.001
Tricuspid annular plane systolic excursion (mm)	22 (20–27)	20 (17–24)	< 0.01
Systolic tricuspid annular velocity (cm/s)	15 (13–18)	14 (12–16)	0.06
RV fractional area change (%)	0.48 (0.43–0.55)	0.45 (0.38–0.53)	0.02
RV/LV end-diastolic areas ratio	0.36 (0.17–0.62)	0.47 (0.18–0.64)	0.37
Systolic pulmonary arterial pressure (mmHg)	27 (20–36)	28 (22–40)	0.45
Cor pulmonale, n (%)	0 (0)	7 (6)	0.04
Significant valvulopathy, n (%)	1 (1)	3 (2)	1.00
Pericardial effusion, n (%)	1 (1)	8 (7)	0.09
Electrocardiogram findings			
* Signs of LV abnormalities, n (%)*	9 (11)	66 (56)	< 0.001
Repolarization abnormalities, n (%)	6 (8)	44 (37)	< 0.001
Inverted T waves, n (%)	5 (6)	32 (30)	< 0.001
ST segment elevation, n (%)	0 (0)	9 (8)	0.01
ST segment depression, n (%)	1 (1)	18 (15)	< 0.01
Pathological Q waves, n (%)	2 (2)	18 (15)	< 0.01
New left branch bundle block, n (%)	3 (4)	10 (8)	0.20
Life-threatening ventricular arrhythmia, n (%)	0 (0)	3 (2)	0.15
Severe bradyarrhythmia, n (%)	0 (0)	5 (4)	0.06
* New-onset atrial arrhythmias, n (%)*	1 (1)	10 (8)	0.03
Atrial fibrillation, n (%)	1 (1)	9 (8)	0.04
Atrial flutter, n (%)	0 (0)	1 (1)	1.00
Atrial tachycardia, n (%)	0 (0)	0 (0)	–
* Signs of RV strain, n (%)*	12 (15)	23 (19)	0.57
Inverted T waves in leads V1-V4, n (%)	2 (2)	13 (11)	0.03
QR pattern in V1, n (%)	0 (0)	2 (2)	0.52
S1Q3T3 pattern, n (%)	6 (8)	6 (5)	0.55
New incomplete or complete right bundle branch, n (%)	5 (6)	9 (8)	0.74

n = 198. Data are expressed as median (interquartile range) or numbers (percentages)

ICU: intensive care unit; TTE: transthoracic echocardiography; PBW: predicted body weight; FiO_2_: inspired oxygen fraction; PaO_2_: partial arterial pressure of oxygen; PaCO_2_: partial arterial pressure of carbon dioxide; LV: left ventricular; VTI: velocity–time integral of the left ventricular outflow tract; E: early peak velocity of transmitral flow with pulsed Doppler; A: atrial peak velocity of transmitral flow with pulsed Doppler; e’: early diastolic peak velocity of the mitral annulus with tissue Doppler imaging; RV: right ventricular; ECG: electrocardiogram

Patients with cardiac injury during ICU stay received more frequently norepinephrine (39 *vs.* 19%, p < 0.01) and were more frequently mechanically ventilated (59 *vs.* 35%, p < 0.01) on ICU admission (Table 3) and had a higher ICU mortality rate than those without (30% *vs*. 9%, p < 0.001) (Table 1). Patients who died in ICU showed more frequently ECG signs of LV abnormalities (53% *vs.* 34%, p = 0.02), new-onset atrial arrhythmias (19% *vs.* 2%, p < 0.001) but not ECG signs of RV strain (21% *vs.* 17%, p = 0.50) on ICU admission. Although no echocardiographic variables were associated with ICU mortality, patients who died in ICU had more frequently LV systolic (27% *vs.* 10%, p = 0.01), LV diastolic dysfunction (51% *vs.* 21%, p < 0.001), cor pulmonale (9% *vs.* 2%, p = 0.04), significant valvulopathy (7% *vs.* 1%, p = 0.03), but not RV systolic dysfunction (29% *vs.* 19%, p = 0.11) or pericardial effusion (7% *vs.* 4%, p = 0.41) on ICU admission.

### Impact of biological sex on cardiac injury

Overall, 147 (74%) patients were men and 51 (26%) were women. Clinical and biological characteristics on ICU admission as well as management and outcomes of patients according to biological sex are summarized in Table 4, Additional file 1: Table S2 and Figure S3. The proportion of patients with cardiac injury during ICU stay was not different between men and women (60% *vs.* 61%, p = 1.00). The delay between the onset of symptoms and ECG and TTE examination did not differ between men and women (8 (7–12) *vs.* 9 (7–12) days, p = 0.43). At TTE examination, ventilatory management, oxygenation and hemodynamic variables did not differ between men and women, to the exception of the respiratory system compliance higher in men than in women (31 (28–40) *vs.* 27 (21–34) mL/cmH_2_O, p = 0.01) (Additional file 1: Table S3).

**Table 4:** Patient characteristics, management and outcomes according to biological sex

Variables	Women (n = 51)	Men (n = 147)	p value
Clinical characteristics			
Age (years)	68 (60–73)	66 (57–73)	0.68
SAPS-2	43 (30–57)	41 (31–59)	0.99
SOFA score on ICU admission	6 (3–10)	7 (3–10)	0.34
Body mass index (kg/m^2^)	29 (26–34)	28 (26–31)	0.24
Obesity, n (%)	24 (47)	54 (38)	0.24
Arterial hypertension, n (%)	26 (51)	77 (52)	0.87
Diabetes mellitus, n (%)	19 (37)	42 (29)	0.29
Dyslipidemia, n (%)	15 (29)	37 (25)	0.58
Coronary artery disease, n (%)	4 (8)	20 (14)	0.33
Stroke, n (%)	3 (6)	6 (4)	0.70
Smokers, n (%)	6 (12)	27 (18)	0.38
Cardiovascular risk factors, n (%)	40 (78)	117 (80)	0.84
Chronic cardiac disease, n (%)	2 (4)	12 (8)	0.52
Chronic respiratory disease, n (%)	5 (10)	6 (4)	0.15
Chronic kidney disease, n (%)	2 (4)	24 (16)	0.03
Neoplasia, n (%)	13 (25)	16 (11)	0.02
Immunosuppression, n (%)	7 (14)	15 (10)	0.61
Renin-Angiotensin System Blockers, n (%)	15 (29)	60 (41)	0.18
Treatments on ICU admission			
Corticosteroids, n (%)	46 (90)	125 (85)	0.48
Tocilizumab, n (%)	14 (27)	32 (22)	0.44
Antiviral drugs, n (%)	2 (4)	9 (6)	0.73
Low-dose thrombophylaxis, n (%)	3 (6)	14 (10)	0.57
Enhanced intermediate-dose thrombophylaxis, n (%)	37 (72)	101 (69)	0.72
Curative anticoagulation, n (%)	11 (22)	41 (28)	0.46
Management during ICU stay			
Neuromuscular blocker agents, n (%)	32 (63)	93 (63)	0.87
Prone positioning, n (%)	27 (53)	87 (59)	0.51
Venovenous ECMO, n (%)	1 (2)	7 (5)	0.68
Renal replacement therapy, n (%)	6 (12)	30 (20)	0.21
Delays and outcomes			
From onset of symptoms to ICU admission ( days)	7 (6–12)	9 (6–11)	0.35
Cardiac injury, n (%)	31 (61)	88 (60)	1.00
Acute respiratory distress syndrome, n (%)	34 (67)	98 (67)	1.00
Pulmonary embolism, n (%)	7 (14)	22 (15)	1.00
Acute kidney injury, n (%)	19 (38)	72 (49)	0.19
Disseminated intravascular coagulation, n (%)	1 (2)	0 (0)	0.26
Duration of invasive mechanical ventilation (days)	11 (6–17)	19 (6–37)	0.04
ICU length of stay (days)	9 (5–15)	12 (6–30)	0.03
ICU mortality rate (n, %)	9 (18)	34 (23)	0.55

n = 198. Data are expressed as median (interquartile range) or numbers (percentages)

ECMO: extracorporeal membrane oxygenation; ICU: intensive care unit; SAPS: simplified acute physiology score; SOFA: sepsis-related organ failure assessment

The proportion of patients with LV systolic dysfunction, LV diastolic dysfunction, RV systolic dysfunction, cor pulmonale, significant valvulopathy or pericardial effusion on ICU admission did not differ between men and women. TTE measurements were overall similar between men and women, to the exception of the velocity–time integral of the LV outflow tract (22 (17–24) *vs.* 23 (19–28) cm, p < 0.01), the E/e’_averaged_ ratio (7.8 (6.4–9.3) *vs.* 8.9 (7.1–10.3), p = 0.03), and the systolic pulmonary arterial pressure (26 (20–33) *vs.* 33 (25–43) mmHg, p < 0.01), which were lower in men than in women (Additional file 1: Table S3). To the exception of ECG signs of LV abnormalities less frequent in men than in women (32% *vs.* 55%, p < 0.01), the proportion of other ECG abnormalities on ICU admission did not differ between men and women (Additional file 1: Table S4).

At multivariate analysis, the presence of cardiovascular risk factors (OR = 2.31; 95%CI (1.06–5.02), p = 0.03) and a medical history of chronic cardiac disease (OR = 8.58; 95%CI (1.01–73.17), p = 0.04) were associated with the occurrence of cardiac injury during ICU stay, whereas biological sex (OR = 0.88; 95%CI (0.42–1.84), p = 0.73) or intubation (OR = 1.86; 95%CI (0.78–4.45), p = 0.16) were not (Table 5).

**Table 5:** Risk factors for occurrence of cardiac injury during ICU stay

Variables	Odds ratio	95% confidence interval	p-value
Cardiovascular risk factors	2.30	(1.06–5.01)	0.03
Chronic cardiac disease	8.49	(1.06–71.86)	0.04
Biological sex	0.88	(0.42–1.84)	0.73
Intubation	1.86	(0.78–4.45)	0.16
Norepinephrine	1.34	(0.50–3.61)	0.56
Leukocytes	1.07	(0.98–1.16)	0.12
Lymphocytes	0.61	(0.34–1.09)	0.09
D-Dimers	1.00	(0.99–1.01)	0.84
C-Reactive Protein	1.00	(0.99–1.01)	0.08
Procalcitonin	0.99	(0.98–1.01)	0.19

Cardiovascular risk factors mean at least one cardiovascular risk factor

n = 198. ICU: intensive care unit

### Impact of biological sex on other clinical outcomes

The proportion of patients with ARDS, pulmonary embolism, acute kidney injury or disseminated intravascular coagulation during ICU stay was not different between men and women (Table 4). While ICU mortality rate did not differ between men and women (23% *vs.* 18%, p = 0.55), duration of invasive mechanical ventilation and ICU length of stay were longer in men than in women (Table 4).

## Discussion

Most critically ill patients with COVID-19 were men and 60% of them experienced cardiac injury during ICU stay. Patients with cardiac injury during ICU stay received more frequently norepinephrine and were more frequently mechanically ventilated on ICU admission and had a higher ICU mortality rate. Multivariate analysis showed cardiovascular risk factors and chronic cardiac disease, but not biological sex, were independently associated with the occurrence of cardiac injury during ICU stay. Biological sex had also no impact on the occurrence during ICU stay of the other clinical outcomes, namely ARDS, pulmonary embolism, acute kidney injury or disseminated intravascular coagulation.

Here, we confirmed that most of COVID-19 patients developed cardiac injury during ICU stay and that a significant proportion of patients had cardiac injury on ICU admission [5]. Patients with cardiac injury during ICU stay had more frequently ECG signs of LV abnormalities, new-onset atrial arrhythmias, LV systolic and diastolic dysfunction as well as RV systolic dysfunction on ICU admission. In agreement with the existing literature, we also found that LV ejection fraction was preserved in most patients with COVID-19 and that RV systolic dysfunction was more frequent than LV systolic dysfunction [5, 23].

We also confirmed that the prognosis of patients with cardiac injury was poorer, regardless of its the definition [5–7, 44]. Patients with cardiac injury who died in ICU showed more frequently ECG signs of LV abnormalities, new-onset atrial arrhythmias LV systolic and diastolic dysfunction but not RV systolic dysfunction on ICU admission. To our knowledge, no study has investigated the potential relationship between ECG abnormalities on ICU admission and mortality in patients with COVID-19 yet. Interestingly, we found no relationship between RV systolic dysfunction and mortality in our cohort, in contrast to other studies which associated RV dysfunction with mortality in patients with COVID-19 [8–11, 45]. This discrepancy may be explained as follow. First, the prognostic value of RV injury in COVID-19 patients may depend on the severity of RV injury, as RV dilation with systolic impairment [10] or acute core pulmonale [8, 9, 11, 45], but not isolated RV dilation or RV dysfunction without RV dilation [9, 10, 45, 46] were found to be independently associated with mortality. Second, the prognostic value of RV injury may also depend on the timing of echocardiography, as we previously showed cardiac injury, including RV dysfunction, may occur later during the ICU stay [5]. Furthermore, in all previous studies that found an association between RV dysfunction and mortality, echocardiographic examinations were not performed on ICU admission but within 72 h of ICU admission [8–11, 45]. Finally, we found no association between any TTE variables on ICU admission and ICU mortality, confirming findings of previous echocardiographic studies which showed that LV and RV strain parameters [47, 48] but not conventional TTE parameters of LV and RV systolic function [46, 48] were associated with prognosis of patients with COVID-19.

Patients with cardiac injury during ICU stay were older, had more frequently cardiovascular risk factors, a more marked lymphopenia and had higher plasmatic levels of inflammatory biomarkers on ICU admission and they also received more frequently norepinephrine and were more frequently mechanically ventilated on ICU admission. Multivariate analysis found cardiovascular risk factors and chronic cardiac disease were independently associated with the occurrence of cardiac injury during ICU stay. Our results therefore suggest that patients at high cardiac risk are most likely to develop cardiac injury during ICU stay, leading to LV and/or RV dysfunction, higher LV filling pressures and therefore a poorer prognosis.

It has been suggested that biological sex may influence the outcomes of patients with COVID-19 with higher severity and organs failure in men [19, 20, 24–26]. This impact of biological sex may be mainly related to the fact that SARS-CoV-2 SARS-CoV-2 entry into host cells is mediated by the host cell’s angiotensin-converting enzyme type 2 (ACE2) receptor [49], and that the ACE2 gene is located in the short arm of the X chromosome [50–52]. ACE2 regulates the renin-angiotensin system and exerts a protective effect against severe organ damage [51] but ACE2 gene polymorphisms were associated with cardiovascular diseases [50]. As the ACE2 gene has the ability to escape inactivation of the X chromosome [53], it confers on women the unique characteristic of possessing two copies of this gene, protecting them against polymorphisms and explaining the differences in ACE2 expression between men and women [50]. Therefore, the existence of a single X chromosome in men has been associated with a significant loss of ACE2 signalling and subsequent cardiovascular disorders compared with two X chromosomes in women, as increased expression of ACE2 in women may be protective against more severe COVID-19 symptoms because rapid viral saturation of ACE2 is less likely to occur [54]. The impact of biological sex may also be related to (i) sex differential immune responses against SARS-CoV-2, as documented for other viral infections, with greater innate and adaptive immune responses in women than in men [55], (ii) genetic factors as many genes with immunomodulatory function are encoded on the X chromosome [56] and (iii) hormonal status [55, 57, 58], with a potential negative effect of low testosterone levels in men in terms of severity of COVID-19 disease and outcomes [57].

Yet, unlike previous studies [19, 20], we did not confirm that biological sex was associated with the occurrence of cardiac injury during ICU stay as well as with other clinical outcomes. A possible explanation may lie in the older age of patients we included in our study, which may have overridden any sex-related difference [19, 20]. It has been shown that the younger the patient, the greater the difference in outcomes between men and women at equivalent ages [20], as the biological sex differences in immunity are most pronounced in pre-menopausal women and age-matched men due to age-related decreases in sex steroid levels [59, 60]. Regarding cardiac injury, this discrepancy may also be explained by the fact we defined cardiac injury by the association of an increase in high-sensitivity troponin T or troponin I levels and newly diagnosed ECG and/or TTE abnormalities [5], when Liu and colleagues defined cardiac injury by an increase in troponin level or newly diagnosed ECG and/or TTE abnormalities [19] and Cho and colleagues defined cardiac injury as myocardial infarction and cardiac arrest only [20].

Finally, we found that plasma levels of inflammatory biomarkers on ICU admission were higher in patient with cardiac injury during ICU stay and in men, and that inflammation had an impact of patient severity, as illustrated by the correlation between plasmatic levels of inflammatory biomarkers and SAPS-2 and SOFA score on ICU admission, confirming the pivotal role of inflammation in disease severity [58, 61]. Nevertheless, although inflammation might be one of the mechanisms of cardiac injury in patients with COVID-19 [62], partly due to H19 depletion, a protein with anti-inflammatory effects, into cardiac endothelium [63], we found no correlation between plasmatic levels of inflammatory biomarkers and the different echocardiographic variables on ICU admission and inflammatory biomarkers were not independently associated with the occurrence of cardiac injury during ICU stay. These results highlight the fact that cardiac injury is not associated with inflammation but rather related to previous cardiac conditions.

We acknowledge some limitations to our study. First, the study period was limited to the first two pandemic waves with low proportion of patients receiving Tocilizumab and no vaccinated patients, while management and SARS-CoV-2 variants have evolved over time. Second, certain cardiac medical histories such as arrhythmia and valvulopathy, as well as home cardiac medications were not specifically collected with the exception to Renin-Angiotensin System Blockers due to the potential interaction between this medication and Sars-CoV-2 infection. Thus, it remains unclear whether patients suffering from chronic conditions develop cardiac injury or whether the severity of the disease and inflammation drive cardiac injury, even if we found in our cohort that cardiovascular risk factors and chronic cardiac disease but not inflammation were independently associated with the occurrence of cardiac injury during ICU stay. Third, no hormonal dosages were performed to confirm the potential role of hormonal status in the impact of biological sex on clinical outcomes. Nevertheless, 73% of women were > 60 years old in our cohort, thus limiting the possibility of exploring this pathophysiological hypothesis. Fourth, data were collected only on ICU admission and therefore, did not necessarily reflect the patient severity during ICU stay and did not capture the dynamics between cardiac injury and its different potentially related factors, including cytokine storm [50], that may occur later during ICU stay. Similarly, ECG and echocardiographic examinations were performed only on ICU admission without longitudinal echocardiographic follow-up during ICU stay. The potential impact of ARDS and mechanical ventilation on hemodynamics and RV function could not therefore be evaluated. Fifth, the potential chronicity of some newly diagnosed ECG and/or echocardiographic abnormalities could not be excluded with certainty as some patients had no or no available results of a previous cardiac evaluation. Sixth, it cannot be ruled out that some of the newly diagnosed cardiac abnormalities were not totally specific to COVID-19 but could also reflect the severity of the disease and be an epiphenomenon of critical illness. Finally, patients were including during the first two pandemic waves when patients were intubated very early, and the use of high-flow nasal canula oxygen therapy was less widespread. Therefore, our findings need to be verified in patients managed during the following pandemic waves.

## Conclusions

Most critically ill patients with COVID-19 were men and experienced cardiac injury during ICU stay. Cardiovascular risk factors and chronic cardiac disease, but not biological sex, were independently associated with the occurrence of cardiac injury during ICU stay and patients with cardiac injury had a higher ICU mortality rate. Biological sex had also no impact on the occurrence during ICU stay of the other clinical outcomes, namely ARDS, pulmonary embolism, acute kidney injury or disseminated intravascular coagulation.

### Supplementary Information

** Additional file 1.** Supplementary tables and figures.

## Data Availability

The datasets used and/or analyzed during the current study are available from the corresponding author on reasonable request.

## Abbreviations

A: Atrial peak velocity of the mitral flow with pulsed Doppler
ACE2: Angiotensin-converting enzyme type 2
ARDS: Acute respiratory distress syndrome
CI: Confidence interval
COVID-19: Coronavirus disease 19
E: Early peak velocity of the mitral flow with pulsed Doppler
e’: Early diastolic peak velocity of the mitral annulus with Tissue Doppler Imaging
ECG: Electrocardiogram
ICU: Intensive care unit
LV: Left ventricular
LVEDA: Left ventricular end-diastolic area
OR: Odds ratio
RV: Right ventricular
RVEDA: Right ventricular end-diastolic area
TTE: Transthoracic echocardiography
