# Supplementary material for: Impact of sex differences on cardiac injury in critically ill patients with COVID-19
Source: Respir Res. 2023 Nov 20;24:292. doi: 10.1186/s12931-023-02581-5 (PMC10662091; doi:10.1186/s12931-023-02581-5)
Supplement: Supplementary file 1 — Additional file 1. Supplementary tables and figures. [file 12931_2023_2581_MOESM1_ESM.docx]

**Impact of sex differences on cardiac injury in critically ill patients with COVID-19**

Mathieu JOZWIAK, MD PhD^1,2,3^ ; Denis DOYEN, MD PhD^3,4^ ; Pierre DENORMANDIE, MD^1,^ ; Antoine GOURY, MD^5^ ; Jonathan MAREY MD^6^ ; Frédéric PENE, MD PhD^1,2^ ; Alain CARIOU, MD PhD^1,2^ ; Jean-Paul MIRA, MD PhD^1,2^ ; Jean DELLAMONICA, MD PhD^3,4^ ; Lee S. NGUYEN MD PhD^1,7^

1 : Service de Médecine Intensive Réanimation, Hôpitaux Universitaires Paris Centre, Hôpital Cochin, Assistance Publique – Hôpitaux de Paris, 27 Rue du Faubourg Saint Jacques, 75014 Paris, France

2 : Université Paris Cité, Paris, France

3 : UR2CA - Unité de Recherche Clinique Côte d'Azur, Université Côte d’Azur, Nice, France

4 : Service de Médecine Intensive Réanimation, Centre Hospitalier Universitaire de Nice, Hôpital l’Archet 1, 151 rue saint Antoine de Ginestière, 06200 Nice, France

5 : Service de Médecine Intensive Réanimation, Centre Hospitalier Universitaire de Reims, Rue du général Koenig, 51092 Reims, France

6 : Unité de soins intensifs pneumologiques, Hôpitaux Universitaires Paris Centre, Hôpital Cochin, Assistance Publique – Hôpitaux de Paris, 27 Rue du Faubourg Saint Jacques, 75014 Paris, France

7 : Recherche et Innovation, Groupe hospitalier privé Ambroise Paré – Hartmann, 48ter Bd Victor Hugo, 92200 Neuilly-sur-Seine, France

***Corresponding author***

Mathieu JOZWIAK, MD, PhD

Hôpital Cochin, APHP

Service de Médecine Intensive Réanimation

27 Rue du Faubourg Saint Jacques, 75014 Paris, France

[jozwiak.m@chu-nice.fr](mailto:jozwiak.m@chu-nice.fr)

***Emails of authors***

Mathieu JOZWIAK : [jozwiak.m@chu-nice.fr](mailto:jozwiak.m@chu-nice.fr)

Denis DOYEN : [doyen.d@chu-nice.fr](mailto:doyen.d@chu-nice.fr)

Pierre DENORMANDIE : [pierre.denormandie@aphp.fr](mailto:pierre.denormandie@aphp.fr)

Antoine GOURY : agoury@chu-reims.fr

Jonathan MAREY : [jonathan.marey@aphp.fr](mailto:jonathan.marey@aphp.fr)

Frédéric PENE : [frederic.pene@aphp.fr](mailto:frederic.pene@aphp.fr)

Alain CARIOU : [alain.cariou@aphp.fr](mailto:alain.cariou@aphp.fr)

Jean-Paul MIRA : [jean-paul.mira@aphp.fr](mailto:jean-paul.mira@aphp.fr)

Jean DELLAMONICA : [dellamonica.j@chu-nice.fr](mailto:dellamonica.j@chu-nice.fr)

Lee S. NGUYEN : [nguyen.lee@icloud.com](mailto:nguyen.lee@icloud.com)

**Additional Tables**

**Table S1. Missing data for each variable in the whole population.**

|  | Patients  (n=198) |
| --- | --- |
| Clinical characteristics |  |
| Age (years) | 0% (198) |
| Biological sex (M/F) | 0% (198) |
| SAPS-2 score | 0% (198) |
| SOFA score | 0% (198) |
| Body mass index (kg/m^2^) | 0% (198) |
| Obesity, n (%) | 0% (198) |
| Arterial hypertension, n (%) | 0% (198) |
| Diabetes mellitus, n (%) | 0% (198) |
| Dyslipidemia, n (%) | 0% (198) |
| Coronary artery disease, n (%) | 0% (198) |
| Stroke, n (%) | 0% (198) |
| Smokers, n (%) | 0% (198) |
| Cardiovascular risk factors, n (%) | 0% (198) |
| Chronic cardiac disease, n (%) | 0% (198) |
| Chronic respiratory disease, n (%) | 0% (198) |
| Chronic kidney disease, n (%) | 0% (198) |
| Neoplasia, n (%) | 0% (198) |
| Immunosuppression, n (%) | 0% (198) |
| Renin-Angiotensin System Blockers, n (%) | 0% (198) |
|  |  |
| Treatments on ICU admission |  |
| Corticosteroids, n (%) | 0% (198) |
| Tocilizumab, n (%) | 0% (198) |
| Antiviral drugs, n (%) | 0% (198) |
| Thrombophylaxis, n (%) | 0% (198) |
|  |  |
| Management during ICU stay | 0% (198) |
| Neuromuscular blocker agents, n (%) | 0% (198) |
| Prone positioning, n (%) | 0% (198) |
| Venovenous ECMO, n (%) | 0% (198) |
| Renal replacement therapy, n (%) | 0% (198) |
|  |  |
| Delays and outcomes |  |
| From onset of symptoms to ICU admission (days) | 0% (198) |
| Cardiac injury, n (%) | 0% (198) |
| Acute respiratory distress syndrome, n (%) | 0% (198) |
| Pulmonary embolism, n (%) | 0% (198) |
| Acute kidney injury, n (%) | 0% (198) |
| Disseminated intravascular coagulation, n (%) | 0% (198) |
|  |  |
| Biological variables |  |
| Leukocytes (x10^9^/L) | 0% (198) |
| Neutrophils (x10^9^/L) | 0% (198) |
| Platelet count (x10^9^/L) | 0% (198) |
| Lymphocytes (x10^9^/L) | 0% (198) |
| Fibrinogen (g/L) | 0% (198) |
| D-Dimers (µg/L) | 1% (196) |
| C-Reactive Protein (mg/L) | 1% (197) |
| Procalcitonin (ng/L) | 3% (191) |
| Ferritin (ng/mL) | 25% (148) |
| Interleukin-6 (pg/mL) | 21% (157) |
| Interleukin-1 (pg/mL) | 40% (119) |
| Troponin T (ng/L) | 0% (98) |
| Troponin I (ng/L) | 0% (100) |
| B-type natriuretic peptide (pg/mL) | 0% (98) |
| N-terminal pro B-type natriuretic peptide (pg/mL) | 0% (100) |
| Potassium (mmol/L) | 0% (198) |
| Magnesium (mmol/L) | 1% (196) |
| Renal clearance (mL/mn) | 0% (198) |
| PaO_2_/FiO_2_ ratio | 0% (198) |
| PaCO_2_ (mmHg) | 0% (198) |
| Arterial blood lactate level (mmol/L) | 0% (198) |
|  |  |
| Variables at TTE examination |  |
| Ventilatory management | 0% (198) |
| Tidal volume (mL/kg of PBW)* | 0% (98) |
| Positive end-expiratory pressure (mmHg)* | 0% (98) |
| Driving pressure (mmHg)* | 0% (98) |
| Respiratory system compliance (mL/cmH2O)* | 0% (98) |
| Hemodynamic variables | 0% (198) |
| Vasopressors and inotropes | 0% (198) |
|  |  |
| Echocardiographic variables |  |
| LV ejection fraction (%) | 0% (198) |
| VTI (cm) | 0% (198) |
| E/A ratio | 8% (182) |
| E/e’_averaged_ | 1% (197) |
| Indexed left atrial volume (mL/m^2^) | 0% (198) |
| Indexed end-diastolic LV volume (mL/m^2^) | 0% (198) |
| Tricuspid annular plane systolic excursion (mm) | 1% (195) |
| Systolic tricuspid annular velocity (cm/s) | 3% (192) |
| RV fractional area change (%) | 2% (193) |
| RV/LV end-diastolic areas ratio | 1% (197) |
| Systolic pulmonary arterial pressure (mmHg) | 35% (129) |
|  |  |
| Electrocardiogram findings |  |
| Signs of LV abnormalities, n (%) | 0% (198) |
| New-onset atrial arrhythmias, n (%) | 0% (198) |
| Signs of RV strain, n (%) | 0% (198) |
|  |  |
| Variables are summarized as percentages of missing data (number of available data).  *Among the 98 patients who were intubated.  Abbreviations: SAPS: simplified acute physiology score; SOFA: sepsis-related organ failure assessment; ICU: intensive care unit; ECMO: extracorporeal membrane oxygenation; FiO2: inspired oxygen fraction; PaO2: partial arterial pressure of oxygen; PaCO2: partial arterial pressure of carbon dioxide; TTE: transthoracic echocardiography; PBW: predicted body weight; LV: left ventricular; VTI: velocity-time integral of the left ventricular outflow tract; E: early peak velocity of transmitral flow with pulsed Doppler; A: atrial peak velocity of transmitral flow with pulsed Doppler; e’: early diastolic peak velocity of the mitral annulus with tissue Doppler imaging; RV: right ventricular.  . | |

| **Table S2. Biological variables on ICU admission according to biological sex.** | | | | | | |
| --- | --- | --- | --- | --- | --- | --- |
| **Variables** | **Women**  **(n=51)** | |  | **Men**  **(n=147)** | | **p value** |
|  |  |  |  |  |  |  |
| Leukocytes (x10^9^/L) | 8.5 | (6.7-11.4) |  | 8.9 | (6.8-12.1) | 0.71 |
|  |  |  |  |  |  |  |
| Neutrophils (x10^9^/L) | 7.4 | (5.3-9.6) |  | 7.4 | (5.6-10.6) | 0.33 |
|  |  |  |  |  |  |  |
| Platelet count (x10^9^/L) | 244 | (160-317) |  | 229 | (180-289) | 0.43 |
|  |  |  |  |  |  |  |
| Lymphocytes (x10^9^/L) | 0.74 | (0.50-1.10) |  | 0.70 | (0.50-0.90) | 0.23 |
|  |  |  |  |  |  |  |
| Fibrinogen (g/L) | 5.4 | (4.7-6.8) |  | 6.7 | (5.4-7.9) | <0.0001 |
|  |  |  |  |  |  |  |
| D-Dimers (µg/L) | 1000 | (660-1869) |  | 1180 | (642-2353) | 0.43 |
|  |  |  |  |  |  |  |
| C-Reactive Protein (mg/L) | 107 | (61-215) |  | 149 | (79-230) | 0.09 |
|  |  |  |  |  |  |  |
| Procalcitonin (ng/L) | 0.21 | (0.08-1.36) |  | 0.31 | (0.15-0.90) | 0.40 |
|  |  |  |  |  |  |  |
| Ferritin (ng/mL) | 902 | (484-1262) |  | 1350 | (801-2616) | 0.001 |
|  |  |  |  |  |  |  |
| Interleukin-6 (pg/mL) | 34 | (10-130) |  | 66 | (26-230) | 0.06 |
|  |  |  |  |  |  |  |
| Interleukin-1 (pg/mL) | 1.00 | (0.32-4.90) |  | 1.82 | (0.32-9.30) | 0.21 |
|  |  |  |  |  |  |  |
| Troponin T (ng/L) | 28 | (12-91) |  | 20 | (13-42) | 0.36 |
|  |  |  |  |  |  |  |
| Troponin I (ng/L) | 17 | (17-27) |  | 23 | (17-53) | 0.11 |
|  |  |  |  |  |  |  |
| B-type natriuretic peptide (pg/mL) | 71 | (49-112) |  | 68 | (29-147) | 0.77 |
|  |  |  |  |  |  |  |
| N-terminal pro B-type natriuretic peptide (pg/mL) | 1052 | (276-7408) |  | 605 | (129-2301) | 0.86 |
|  |  |  |  |  |  |  |
| Potassium (mmol/L) | 4.0 | (3.5-4.3) |  | 4.1 | (3.8-4.5) | 0.03 |
|  |  |  |  |  |  |  |
| Magnesium (mmol/L) | 0.84 | (0.75-1.00) |  | 0.94 | (0.85-1.04) | <0.01 |
|  |  |  |  |  |  |  |
| Renal clearance (mL/mn) | 90 | (56-98) |  | 87 | (54-104) | 0.89 |
|  |  |  |  |  |  |  |
| Arterial blood lactate level (mmol/L) | 1.1 | (0.9-1.5) |  | 1.1 | (0.9-1.5) | 0.88 |
|  |  |  |  |  |  |  |
| n=198. Data are expressed as median (interquartile range).  ICU: intensive care unit. | | | | | | |

| **Table S3. Echocardiographic findings on ICU admission according to biological sex.** | | | | | | |
| --- | --- | --- | --- | --- | --- | --- |
| **Variables** | **Women**  **(n=51)** | |  | **Men**  **(n=147)** | | **p value** |
|  |  |  |  |  |  |  |
| **Ventilatory management at TTE examination** |  |  |  |  |  |  |
|  |  |  |  |  |  |  |
| High-flow nasal canula oxygen therapy, n (%) | 27 | (53) |  | 61 | (42) | 0.19 |
|  |  |  |  |  |  |  |
| Non-invasive ventilation, n (%) | 3 | (6) |  | 9 | (6) | 1.00 |
|  |  |  |  |  |  |  |
| Intubation, n (%) | 21 | (41) |  | 77 | (52) | 0.23 |
|  |  |  |  |  |  |  |
| Tidal volume (mL/kg of PBW) | 6.1 | (5.7-6.3) |  | 6.0 | (5.7-6.2) | 0.40 |
|  |  |  |  |  |  |  |
| Positive end-expiratory pressure (mmHg) | 12 | (8-13) |  | 12 | (10-14) | 0.20 |
|  |  |  |  |  |  |  |
| Driving pressure (mmHg) | 13 | (11-15) |  | 13 | (11-14) | 0.93 |
|  |  |  |  |  |  |  |
| Respiratory system compliance (mL/cmH2O) | 27 | (21-34) |  | 31 | (28-40) | 0.01 |
|  |  |  |  |  |  |  |
| **Oxygenation variables at TTE examination** |  |  |  |  |  |  |
|  |  |  |  |  |  |  |
| PaO_2_/FiO_2_ ratio | 121 | (87-165) |  | 125 | (81-180) | 0.86 |
|  |  |  |  |  |  |  |
| PaCO_2_ (mmHg) | 37 | (32-43) |  | 37 | (33-42) | 0.88 |
|  |  |  |  |  |  |  |
| **Hemodynamic variables at TTE examination** |  |  |  |  |  |  |
|  |  |  |  |  |  |  |
| Heart rate (beats per minute) | 75 | (68-85) |  | 78 | (67-90) | 0.50 |
|  |  |  |  |  |  |  |
| Systolic arterial pressure (mmHg) | 124 | (116-136) |  | 122 | (109-132) | 0.34 |
|  |  |  |  |  |  |  |
| Diastolic arterial pressure (mmHg) | 63 | (57-72) |  | 63 | (56-73) | 0.88 |
|  |  |  |  |  |  |  |
| Mean arterial pressure (mmHg) | 87 | (77-95) |  | 81 | (74-93) | 0.10 |
|  |  |  |  |  |  |  |
| Norepinephrine, n (%) | 17 | (33) |  | 44 | (30) | 0.73 |
|  |  |  |  |  |  |  |
| Norepinephrine dosage (µg/kg/min) | 0.12 | (0.05-0.28) |  | 0.17 | (0.10-0.31) | 0.18 |
|  |  |  |  |  |  |  |
| Dobutamine, n (%) | 0 | (0) |  | 1 | (0.5) | NA |
|  |  |  |  |  |  |  |
| Dobutamine dosage (µg/kg/min) | 0 | (0-0) |  | 5 | (5-5) | NA |
|  |  |  |  |  |  |  |
| **TTE variables** |  |  |  |  |  |  |
|  |  |  |  |  |  |  |
| *LV systolic dysfunction, n (%)* | 8 | (16) |  | 19 | (13) | 0.64 |
|  |  |  |  |  |  |  |
| LV ejection fraction (%) | 64 | (54-69)  ) |  | 61 | (55-67) | 0.53 |
|  |  |  |  |  |  |  |
| VTI (cm) | 23 | (19-28) |  | 22 | (17-24) | <0.01 |
|  |  |  |  |  |  |  |
| Segmental wall motion abnormality, n (%) | 3 | (6) |  | 21 | (14) | 0.14 |
|  |  |  |  |  |  |  |
| *LV diastolic dysfunction, n (%)* | 18 | (35) |  | 36 | (24) | 0.15 |
|  |  |  |  |  |  |  |
| E/A ratio | 0.93 | (0.79-1.14) |  | 0.98 | (0.79-1.17) | 0.62 |
|  |  |  |  |  |  |  |
| e’_lateral_ (cm/s) | 9.5 | (8.0-12.0) |  | 10.0 | (8.0-12.0) | 0.41 |
|  |  |  |  |  |  |  |
| e’_septal_ (cm/s) | 8.0 | (6.4-9.0) |  | 8.0 | (6.4-9.5) | 1.00 |
|  |  |  |  |  |  |  |
| E/e’_averaged_ | 8.9 | (7.1-10.3) |  | 7.8 | (6.4-9.3) | 0.03 |
|  |  |  |  |  |  |  |
| Indexed left atrial volume (mL/m^2^) | 21 | (16-29)  ) |  | 22 | (17-31) | 0.21 |
|  |  |  |  |  |  |  |
| LV hypertrophy, n (%) | 15 | (29) |  | 42 | (29) | 1.00 |
|  |  |  |  |  |  |  |
| Indexed end-diastolic LV volume (mL/m^2^) | 42 | (33-49) |  | 46 | (35-56) | 0.13 |
|  |  |  |  |  |  |  |
| *RV systolic dysfunction, n (%)* | 10 | (20) |  | 32 | (22) | 0.84 |
|  |  |  |  |  |  |  |
| Tricuspid annular plane systolic excursion (mm) | 21 | (19-24) |  | 21 | (18-26) | 1.00 |
|  |  |  |  |  |  |  |
| Systolic tricuspid annular velocity (cm/s) | 15 | (14-17) |  | 14 | (12-17) | 0.10 |
|  |  |  |  |  |  |  |
| RV fractional area change (%) | 0.46 | (0.42-0.53) |  | 0.45 | (0.40-0.54) | 0.86 |
|  |  |  |  |  |  |  |
| RV/LV end-diastolic areas ratio | 0.47 | (0.17-0.62) |  | 0.41 | (0.18-0.64) | 0.88 |
|  |  |  |  |  |  |  |
| Systolic pulmonary arterial pressure (mmHg) | 33 | (25-43) |  | 26 | (20-33) | <0.01 |
|  |  |  |  |  |  |  |
| Cor pulmonale, n (%) | 0 | (0) |  | 7 | (5) | 0.19 |
|  |  |  |  |  |  |  |
| Significant valvulopathy, n (%) | 1 | (2) |  | 3 | (2) | 1.00 |
|  |  |  |  |  |  |  |
| Pericardial effusion, n (%) | 1 | (2) |  | 8 | (5) | 0.28 |
|  |  |  |  |  |  |  |
| n=198. Data are expressed as median (interquartile range) or numbers (percentages).  ICU: intensive care unit; TTE: transthoracic echocardiography; PBW: predicted body weight; FiO_2_: inspired oxygen fraction; PaO_2_: partial arterial pressure of oxygen; PaCO_2_: partial arterial pressure of carbon dioxide; LV: left ventricular; VTI: velocity-time integral of the left ventricular outflow tract; E: early peak velocity of transmitral flow with pulsed Doppler; A: atrial peak velocity of transmitral flow with pulsed Doppler; e’: early diastolic peak velocity of the mitral annulus with tissue Doppler imaging; RV: right ventricular. | | | | | | |

| **Table S4. Electrocardiogram findings on ICU admission according to biological sex.** | | | | | | |
| --- | --- | --- | --- | --- | --- | --- |
| **Variables** | **Women**  **(n=51)** | |  | **Men**  **(n=147)** | | **p value** |
|  |  |  |  |  |  |  |
| **Signs of LV abnormalities, n (%)** | 28 | (55) |  | 47 | (32) | <0.01 |
|  |  |  |  |  |  |  |
| Repolarization abnormalities, n (%) | 17 | (33) |  | 33 | (22) | 0.14 |
|  |  |  |  |  |  |  |
| *Inverted T waves, n (%)*  *ST segment depression, n (%)* | 11 | (22) |  | 26 | (18) | 0.54 |
|  |  |  |  |  |  |  |
| *ST segment elevation, n (%)* | 4 | (8) |  | 5 | (3) | 0.24 |
|  |  |  |  |  |  |  |
| *ST segment depression, n (%)* | 4 | (8) |  | 15 | (10) | 0.79 |
|  |  |  |  |  |  |  |
| Pathological Q waves, n (%) | 8 | (16) |  | 12 | (8) | 0.17 |
|  |  |  |  |  |  |  |
| New left branch bundle block, n (%) | 4 | (8) |  | 9 | (6) | 0.74 |
|  |  |  |  |  |  |  |
| Life-threatening ventricular arrhythmia, n (%) | 1 | (2) |  | 2 | (1) | 1.00 |
|  |  |  |  |  |  |  |
| Severe bradyarrhythmia, n (%) | 3 | (6) |  | 2 | (1) | 0.11 |
|  |  |  |  |  |  |  |
| **New-onset atrial arrhythmias, n (%)** | 3 | (6) |  | 8 | (5) | 1.00 |
|  |  |  |  |  |  |  |
| Atrial fibrillation, n (%) | 3 | (6) |  | 7 | (5) | 0.72 |
|  |  |  |  |  |  |  |
| Atrial flutter, n (%) | 0 | (0) |  | 1 | (1) | 1.00 |
|  |  |  |  |  |  |  |
| Atrial tachycardia, n (%) | 0 | (0) |  | 0 | (0) | - |
|  |  |  |  |  |  |  |
| **Signs of RV strain, n (%)** | 8 | (16) |  | 27 | (18) | 0.83 |
|  |  |  |  |  |  |  |
| Inverted T waves in leads V1-V4, n (%) | 5 | (10) |  | 10 | (7) | 0.54 |
|  |  |  |  |  |  |  |
| QR pattern in V1, n (%) | 0 | (0) |  | 2 | (1) | 1.00 |
|  |  |  |  |  |  |  |
| S1Q3T3 pattern, n (%) | 1 | (2) |  | 11 | (7) | 0.30 |
|  |  |  |  |  |  |  |
| New incomplete or complete right bundle branch, n (%) | 4 | (8) |  | 10 | (7) | 0.76 |
|  |  |  |  |  |  |  |
| n=198. Data are expressed as numbers (percentages).  ICU: intensive care unit; LV: left ventricular; RV: right ventricular. | | | | | | |

**Additional Figures**

**Figure S1**

Flowchart of patients with and without cardiac injury during intensive care unit (ICU) stay. ECG: electrocardiogram; TTE: transthoracic echocardiography.

**Figure S2**

Correlation matrix of echocardiographic variables and plasmatic levels of inflammatory biomarkers on intensive care unit admission. Spearman’s correlations are computed amongst all variables. Positive correlations are represented by red squares and negative correlations by blue squares. Darker colors represent higher correlation coefficient. NT-proBNP: N-terminal pro B-type natriuretic peptide; sPAP: systolic pulmonary artery pressure; RVFAC: right ventricular fractional area change; TAPSE: tricuspid annular plane systolic excursion; S_tric_: systolic tricuspid annular velocity E: early peak velocity of the mitral flow with pulsed Doppler; A: atrial peak velocity of the mitral flow with pulsed Doppler; e’: early diastolic peak velocity of the mitral annulus with Tissue Doppler Imaging; LVEF: left ventricular ejection fraction.

**Figure S3**

Plasmatic levels of inflammatory biomarkers on intensive care unit admission in men (n=147, blue line) and women (n=51, red line). Variables are expressed as median and interquartile range.

**Figure S2**
